# *OsNAC45* is Involved in ABA Response and Salt Tolerance in Rice

**DOI:** 10.1186/s12284-020-00440-1

**Published:** 2020-12-07

**Authors:** Xiang Zhang, Yan Long, Jingjing Huang, Jixing Xia

**Affiliations:** State Key Laboratory for Conservation and Utilization of Subtropical Agro-bioresources, College of Life Science and Technology, Guangxi University, Nanning, 530004 China

**Keywords:** OsNAC45, Rice, Salinity, Salt tolerance, ABA

## Abstract

**Background:**

Salt stress threatens crop yields all over the world. Many NAC transcription factors have been reported to be involved in different abiotic stress responses, but it remains unclear how loss of these transcription factors alters the transcriptomes of plants. Previous reports have demonstrated that overexpression of *OsNAC45* enhances salt and drought tolerance in rice, and that OsNAC45 may regulate the expression of two specific genes, *OsPM1* and *OsLEA3–1*.

**Results:**

Here, we found that ABA repressed, and NaCl promoted, the expression of *OsNAC45* in roots. Immunostaining showed that OsNAC45 was localized in all root cells and was mainly expressed in the stele. Loss of *OsNAC45* decreased the sensitivity of rice plants to ABA and over-expressing this gene had the opposite effect, which demonstrated that *OsNAC45* played an important role during ABA signal responses. Knockout of *OsNAC45* also resulted in more ROS accumulation in roots and increased sensitivity of rice to salt stress. Transcriptome sequencing assay found that thousands of genes were differently expressed in *OsNAC45*-knockout plants. Most of the down-regulated genes participated in plant stress responses. Quantitative real time RT-PCR suggested that seven genes may be regulated by *OsNAC45* including *OsCYP89G1*, *OsDREB1F*, *OsEREBP2*, *OsERF104*, *OsPM1, OsSAMDC2,* and *OsSIK1*.

**Conclusions:**

These results indicate that *OsNAC45* plays vital roles in ABA signal responses and salt tolerance in rice. Further characterization of this gene may help us understand ABA signal pathway and breed rice plants that are more tolerant to salt stress.

**Supplementary Information:**

The online version contains supplementary material available at 10.1186/s12284-020-00440-1.

## Background

Rice is one of the world’s most important crop plants and provides daily sustenance for half of the global human population. However, natural stresses including salinity, inappropriate temperatures or drought cause plant growth retardation and thus reduce crop yields (Sachs and Ho 1986; Zhu 2002; VanWallendael et al. 2019). Salt stress affects crops all over the world; high salinity causes osmotic stress and unbalances ionic homeostasis, leading to growth retardation and decreased agricultural productivity (Zhu 2002; Ismail and Horie 2017). To elucidate how plant responds to these abiotic stresses, numerous stress-related genes have been identified and well-studied. Many of these genes are transcription factors – such as, AP2/EREBP (Riechmann and Meyerowitz 1998), DREB (Liu et al. 1998), bZIP (Kim et al. 2004), MYB (Dai et al. 2007) and NAC (Hu et al. 2006) – that also participate in plant development, signal transduction and responses to environmental stimuli. Changing the expression levels of individual transcription factors in plants may alter the expression profiles of many downstream genes and, therefore, may affect the plants’ tolerance to environmental stresses (Tang et al. 2012; Shim et al. 2018; Yang et al. 2019).

The NAC (NAM, ATAF1/2, CUC2) family is one of the largest families of stress-responsive transcription factors with 117 and 151 predicted members in *Arabidopsis* and rice (Nuruzzaman et al. 2010), respectively. The family appears to be unique to plants (Puranik et al. 2012). The NAC transcription factors have a highly conserved DNA binding domain located at the N-terminal region, while the C-terminal transcriptional activation domains are highly divergent.

The NAM domain was first identified in petunia, and then the ATAF1/2 and CUC2 domains were reported in *Arabidopsis* (Souer et al. 1996; Aida et al. 1997; Dai et al. 2007). Previous studies have demonstrated that NAC transcription factors participate in organ development. For example, NAM mediates the formation of embryos and flowers in petunia (Souer et al. 1996). Loss of *CUC1* and *CUC2* causes the cotyledons of *Arabidopsis* seedlings to curl like a cup (Aida et al. 1997). Another *NAC* gene from *Arabidopsis*, *AtNAC1*, mediates auxin signaling to regulate lateral root growth in seedlings (Xie et al. 2000). Other reports suggest that NAC genes also play vital role in leaf senescence, phytohormone signal transduction and stress responses (Sakuraba et al. 2015; Mao et al. 2017).

ATAF2 directly binds to the promoter region of NIT2 (Nitrilase 2), thereby mediating auxin biosynthesis in *Arabidopsis* (Huh et al. 2012). Overexpression of *OsNAC2* promotes ABA biosynthesis and suppresses ABA catabolic reactions, which increase the ABA content of rice plants (Mao et al. 2017). Overexpressing *SNAC1* (Stress-responsive *NAC* 1) increases the survival rate of transgenic rice during drought or salt stress (Hu et al. 2006). SNAC2, which was isolated from upland rice IRA109, increases rice resistance to salt or cold stress (Hu et al. 2008). PEG, ABA, NaCl and H_2_O_2_ have been shown to induce the expression of another NAC transcription factor in rice, *ONAC066* (Yuan et al. 2019)*,* which promotes drought and oxidative stress resistance phenotypes.

It is widely accepted that the phytohormone abscisic acid (ABA) plays important roles in plant abiotic stress responses (Zhu 2002). The expression levels of *SNAC1*, *SNAC2*/*OsNAC6*, *OsNAC5*, *OsNAC10*, *OsNAC14*, *ONAC022* and *ONAC066* increase under ABA treatment (Hu et al. 2006; Nakashima et al. 2007; Hu et al. 2008; Sperotto et al. 2009; Jeong et al. 2010; Hong et al. 2016; Shim et al. 2018; Yuan et al. 2019). *SNAC1*, *SNAC2*, *OsNAC5*, *OsNAC10*, *OsNAC14* and *ONAC066* increase drought tolerance in rice (Hu et al. 2006; Hu et al. 2008; Jeong et al. 2010; Jeong et al. 2013; Yuan et al. 2019) and *SNAC1*, *OsNAC5* and *OsNAC6* have been shown to enhance the survival of transgenic plants under salt stress (Hu et al. 2006; Nakashima et al. 2007; Takasaki et al. 2010).

ABA also negatively regulates seed germination (Nambara and Marion-Poll 2005). Several stress-related NAC genes including *SNAC1*, *SNAC2*, *OsNAC52* and *ONAC066* have been shown to increase the sensitivity of plants to ABA (Hu et al. 2006; Hu et al. 2008; Gao et al. 2010; Yuan et al. 2019). The expression of *RD26*, a NAC transcription factor isolated from dehydrated *Arabidopsis*, is induced by drought and ABA treatment (Fujita et al. 2004). Transgenic *Arabidopsis* plants overexpressing *RD26* were also found to be more sensitive to ABA treatment than WT plants.

Previous studies reported that overexpressing *OsNAC45* enhances salt and drought tolerance, and decreases cold tolerance, in rice, and that OsNAC45 may regulate the expression of two genes known as *OsPM1* and *OsLEA3–1* (Zheng et al. 2009; Yu et al. 2018). However, the phenotypes of *OsNAC45*-knockout rice under salt and ABA treatment were not investigated. In this paper, we investigated the phenotypes of transgenic rice seedlings that overexpress *OsNAC45*, or have lost the function of this gene, under ABA and NaCl treatment. We also analyzed the transcriptomes of *OsNAC45*-knockout and WT plants under normal growth conditions and high salinity. Our results showed that *OsNAC45* positively regulates ABA signal pathway and is required for salt tolerance in rice.

## Results

### Expression and Transcriptional Activation Analysis of *OsNAC45*

**Fig. 1:**
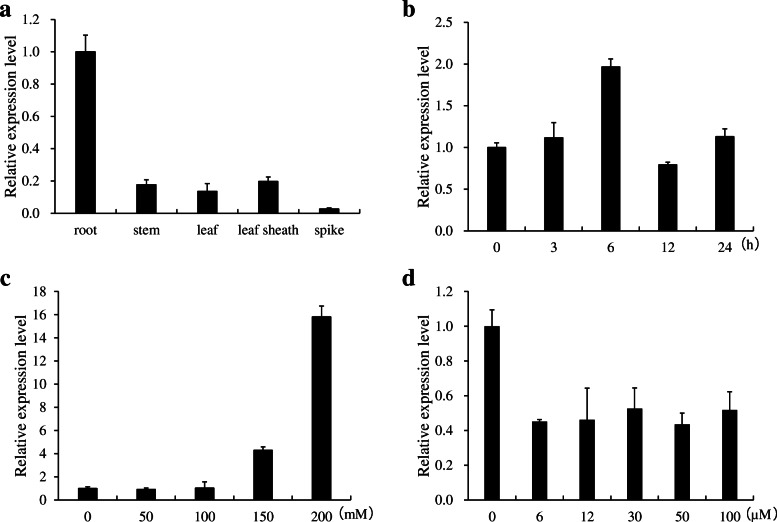
Expression patterns of *OsNAC45*. **a** Expression of *OsNAC45* in various tissues of rice cultivar Nipponbare. **b** Time-course expression pattern of *OsNAC45* in rice roots. Seedlings were exposed to 100 mM NaCl for 3, 6, 12 or 24 h. **c** Dose-dependent expression of *OsNAC45* in rice roots during salt treatment. Seedlings were exposed to different concentrations of NaCl for 12 h. **d** Dose-dependent expression of *OsNAC45* in rice roots during ABA treatment. Seedlings were exposed to different concentrations of ABA for 6 h. Data are means ± SD of three biological replicates

To determine the tissue specificity of *OsNAC45* expression in rice, different tissues (root, stem, leaf, leaf sheath, spike) of WT plants were harvested and the expression of *OsNAC45* was detected by quantitative real time RT-PCR (qRT-PCR). *OsNAC45* was expressed in all the tissues we examined, with the highest level of expression in the roots, and the lowest level of expression in spikes (Fig. 1a).

Since most NAC transcription factors mediated abiotic stress responses, we investigated the expression level of *OsNAC45* during salt and ABA treatments. Time gradient experiments showed that the expression level of *OsNAC45* increased under salt treatment and reached the maximum level at 6 h (Fig. 1b). Dose-dependent experiments demonstrated that the expression level of *OsNAC45* was positively correlated with the concentration of NaCl (Fig. 1c). Although the expression level of *OsNAC45* decreased under ABA treatment, this effect was not dose-dependent (Fig. 1d).

Next, we fused the coding region of *OsNAC45* to the DNA-binding domain of GAL4 (pGBK-OsNAC45) to examine the transcriptional activity of OsNAC45 in yeast. Cells transformed with the pGBK-OsNAC45 vector grew normally on SD/Trp- and SD/Trp−/His−/Ade- plates, while the negative control pGBKT7 was only able to grow on SD/Trp- plates (Additional file 1: Fig. S1). These results suggested that OsNAC45 activates transcription of genes in the yeast cells.

### Subcellular and Cellular Localization of OsNAC45

To determine the subcellular localization of OsNAC45 in rice cells, *GFP* and a *OsNAC45-GFP* fusion construct were introduced into rice protoplasts with the nuclear marker *OsGhd7-RFP*. The fluorescence signals of OsNAC45 overlapped nicely with the nuclear marker while fluorescence signals of the control vector (35S: GFP) were distributed in the whole cell (Additional file 1: Fig. S2).

**Fig. 2:**
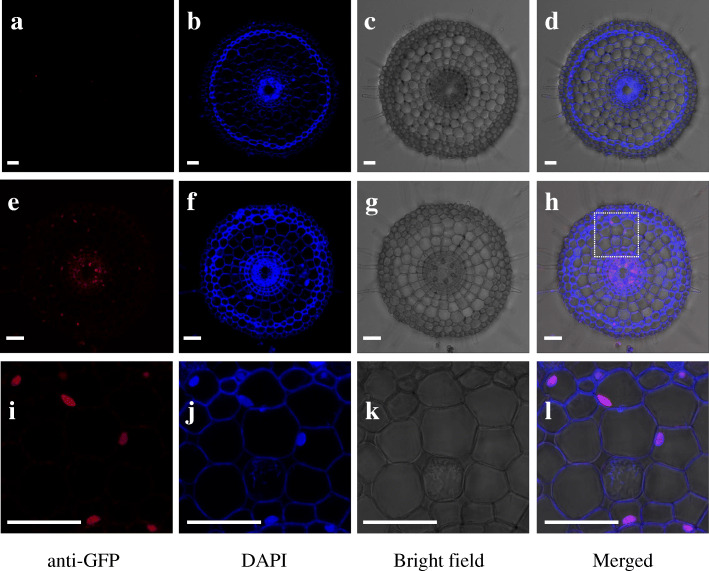
Subcellular and cellular localization of OsNAC45. **a**-**d** Immunostaining of the roots of Nipponbare using the anti-GFP antibody. **e**-**h** Immunostaining of the roots of Pro*OsNAC45-OsNAC45-GFP* transgenic plants. **i**-**l** High-magnification images of dotted part in (**h**). Red color indicates the signal of the anti-GFP antibody, blue color indicates autofluorescence of cell wall and nuclei stained with DAPI. Scale bar = 20 μm

We also examined the cellular localization of OsNAC45 by immunostaining of *ProOsNAC45*-*OsNAC45*-*GFP* transgenic rice. DAPI was used as a nuclear stain. Antibodies against GFP (red signal) co-stained with DAPI, showing that OsNAC45 was localized in nucleus of the rice roots (Fig. 2e-l). This was consistent with our observations in rice protoplast cells. Immunostaining staining also showed that expression level of OsNAC45 was higher in the stele than in other root cell layers (Fig. 2e-h). No signal was observed in the wild-type root (Fig. 2a-d), indicating that antibody against GFP is specific.

### ABA Regulates the Germination and Growth of Young *OsNAC45*-Transgenic Rice Seedlings

Since exogenous ABA repressed the expression of *OsNAC45*(Fig. 1d), it is possible that altering *OsNAC45* expression in rice may affect the plants’ sensitivity to ABA. To confirm this hypothesis, we generated *OsNAC45*-knockout (*osnac45–14*, *osnac45–15*; Additional file 1: Fig. S3) lines using the CRISPR/Cas9 method, and generated *OsNAC45*-overexpression (OE-10, OE-13) lines in which the *OsNAC45* gene was driven by the maize ubiquitin promoter (for the expression levels of *OsNAC45* in the overexpression lines see Additional file 1: Fig. S4).

**Fig. 3:**
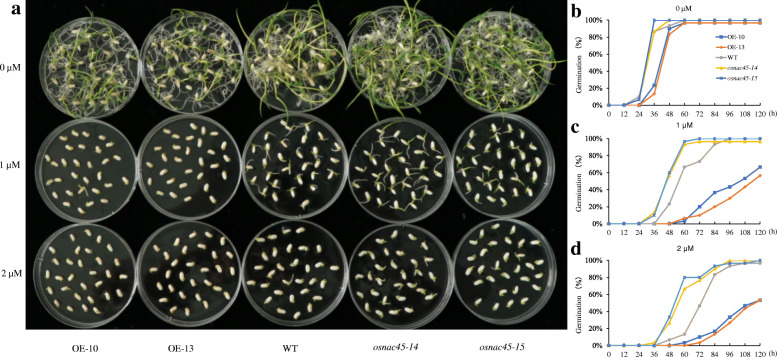
Germination of *OsNAC45*-knockout (*osnac45–14*, *osnac45–15*) and *OsNAC45*-overexpression (OE-10, OE-13) lines under different concentrations of ABA. **a** Germination performance of Nipponbare, *osnac45* and *OsNAC45*-overexpressing seeds after 5 days on 1/2-strength MS medium containing 0, 1, and 2 μM ABA. Germination rates of Nipponbare, *osnac45* and overexpression seeds on 1/2-strength MS medium containing 0 μM ABA (**b**), 1 μM ABA (**c**), 2 μM ABA (**d**)

First, we compared the ABA sensitivity of the transgenic plants with the WT. In the absence of ABA, the germination rates of the transgenic lines were similar to those of the WT lines (Fig. 3a). However, the seeds of the overexpression lines germinated approximately 12 h later than the WT seeds (Fig. 3b). After 1 μM ABA treatment, the seeds of the *osnac45* lines and the WT had nearly all germinated within 72 and 96 h, respectively. However, at 120 h the germination rates of the overexpression lines had only reached 65% (Fig. 3c). When treated with 2 μM exogenous ABA, nearly all the seeds of the *osnac45* and WT lines had germinated within 96 and 108 h, respectively. However, only approximately half of the seeds of the overexpression lines had germinated by 120 h (Fig. 3d).

**Fig. 4:**
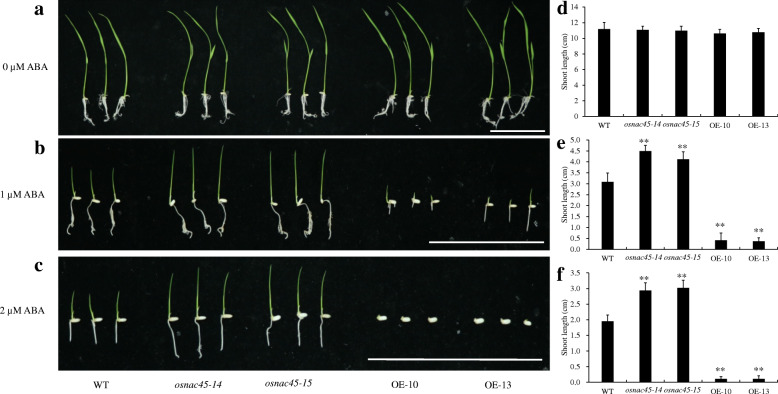
Early growth of *OsNAC45*-knockout (*osnac45–14*, *osnac45–15*) and *OsNAC45*-overexpression (OE-10, OE-13) line seedlings under different concentrations of ABA. Performance of Nipponbare, *osnac45* and OE seedlings 5 days after being transferred to 1/2-strength MS medium containing 0 μM ABA (**a**), 1 μM ABA (**b**), 2 μM ABA (**c**). All seeds were germinated on 1/2-strength MS medium without ABA for 2 days before the ABA treatment. Scale bar = 10 cm. **d** Shoot length of Nipponbare, *osnac45* and OE seedlings after 5 days on 1/2-strength MS medium containing 0 μM ABA (**d**), 1 μM ABA (**e**), 2 μM ABA (**f**). The data represent the means ± SD (*n* ≥ 10 each), **P < 0.01 by the Student’s t test

To further investigate the relationship between *OsNAC45* and exogenous ABA, a post-germination assay was performed. Seeds were placed on 1/2 MS medium for 2 days to germinate and then seedlings with a similar size were transferred to 1/2 MS medium containing different concentrations of ABA. The growth of the *osnac45* and *OsNAC45*-overexpressed lines on the 1/2 MS medium were similar to that of the WT (Fig. 4a, d). In the presence of 1 or 2 μM ABA, the shoots of the WT plants were shorter than those of the *osnac45* lines, but longer than the shoots of the overexpression lines (Fig. 4b, c, e, f). The results from the germination and post-germination assays suggest that overexpressing *OsNAC45* increases the sensitivity of rice plants to ABA treatment and loss of *OsNAC45* has the opposite effect.

### Loss of *OsNAC45* Decreases the Salt Tolerance of Transgenic Rice

**Fig. 5:**
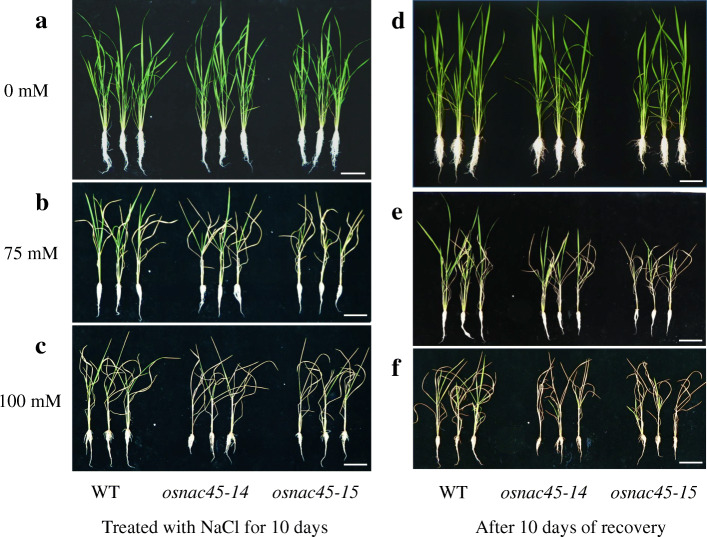
Loss of *OsNAC45* decreases salt tolerance in rice. Performance of Nipponbare and *osnac45* lines treated with 0 mM (**a**), 75 mM (**b**), 100 mM or (**c**) NaCl for 10 days. Performance of Nipponbare and *osnac45* lines that were allowed to recover for 10 days after the 10-day treatment with 0 mM (**d**), 75 mM (**e**) or100 mM (**f**) NaCl. Scale bar = 10 cm. The experiment was repeated three times and similar results were obtained

**Fig. 6:**
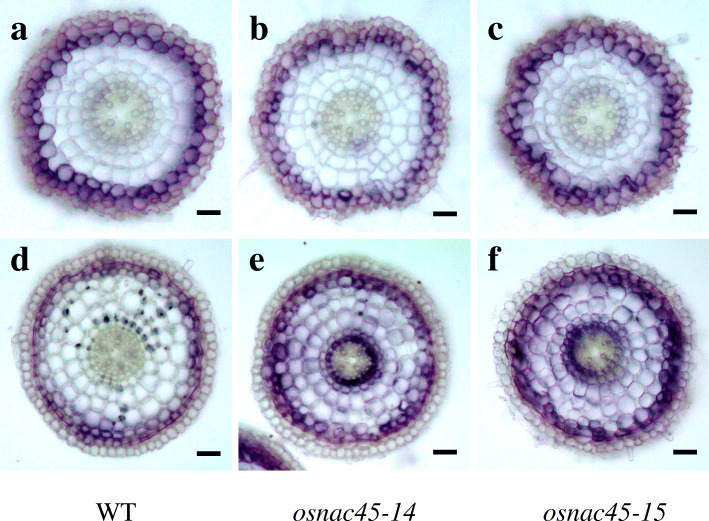
NBT staining in the roots of 5-d-old WT and *osnac45* seedlings under normal and salt conditions. **a-c** Cross section of WT, *osnac45–14* and *osnac45–15* under normal condition, respectively. **d-f** Cross section of WT, *osnac45–14* and *osnac45–15* under salt condition, respectively. Seedlings were treated with 100 mM NaCl for 24 h. Scale bar = 20 μm

Seedlings of the *osnac45* lines and the WT control were exposed to different concentrations of NaCl to determine their ability to tolerate salt stress. In the absence of NaCl, the growth of the *osnac45* lines was similar to that of the WT (Fig. 5a, d). However, when treated with 75 or 100 mM NaCl for 10 days, the *osnac45* lines were smaller and had more withered leaves compared with the WT (Fig. 5b, c). After a 10-day recovery period, the *osnac45* lines had fewer green leaves compared with the WT lines (Fig. 5e, f). Under normal conditions, the dry weights of the *osnac45* lines and the WT plants were similar (Additional file 1: Fig. S5). After salt treatment the WT plants had greater root and shoot dry weights than the *osnac45* lines (Additional file 1: Fig. S5). Furthermore, mineral analysis showed that there was no obvious difference in tissue Na^+^ and K^+^ concentration between WT and *osnac45* lines under normal or salt conditions (Additional file 1: Fig. S6). On the other hand, we also compared the accumulation of reactive oxygen species (ROS) in roots between WT and *osnac45* lines with the nitroblue tetrazolium (NBT) staining. At the root tip region (5 mm), the cross section of both WT and *osnac45* lines exhibited similar intense staining under normal condition (Fig. 6a-c). Under salt conditions, the staining of *osnac45* lines showed more intense while that of WT became weaker compared to the normal conditions, indicating that MT lines accumulated more ROS in roots after salt treatment (Fig. 6d-f). These results suggest that reduced salt tolerance of *osnac45* lines may be caused by more accumulation of ROS, not Na^+^ and K^+^ in rice.

### Loss of *OsNAC45* Alters the Expression Profiles of Many Genes in Rice

**Fig. 7:**
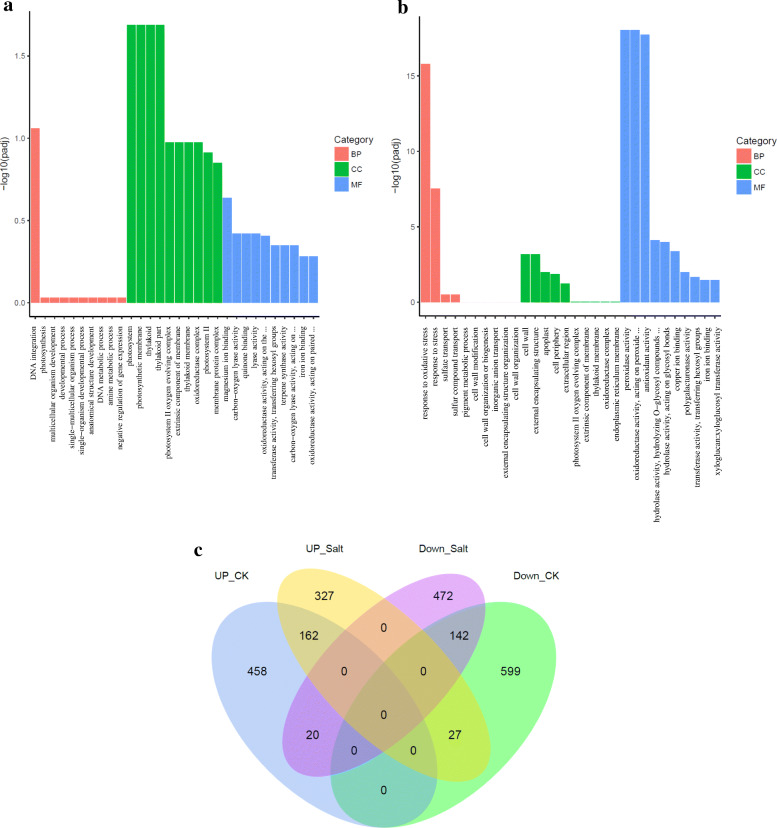
Transcriptome analysis of *OsNAC45*-regulated genes. Classification of up-regulated genes (**a**) and down-regulated genes (**b**) in *osnac45* (MT) transgenic plants compared with the WT under normal conditions. **c** Venn diagram of the numbers of genes that are regulated by OsNAC45 in the MT and WT lines. UP_CK: the up-regulated genes in the MT compared with the WT under normal conditions (fold-change > 2). UP_SALT: the up-regulated genes in the MT compared with the WT in high salinity (fold-change > 2). DOWN_SALT: the down-regulated genes in the MT with the WT in high salinity (fold-change < 0.5). DOWN_CK: the down-regulated genes in the MT compared with the WT under normal conditions (fold-change < 0.5). BP: biological process. CC: cellular component. MF: molecular function. Three biological replicates (*n* = 3) were performed for each treatment

To further investigate the role of *OsNAC45* in rice under both normal and salt conditions, the transcriptomes of Nipponbare (WT) and mutant *osnac45* (MT) rice roots were analyzed using a high-throughput RNA-seq assay. The expression profiles of many genes were significantly different in the MT and WT lines under both normal and salt conditions (Additional file 2). Under normal conditions, 2414 genes were up-regulated and 2746 genes were down-regulated in the MT plants compared with the WT (Additional file 1: Fig. S7a). The up-regulated genes are mainly involved in DNA integration, photosystem, photosynthetic membrane, thylakoid components (Fig. 7a). The down-regulated genes are mainly involved in responses to oxidative stress, stress responses, peroxidase activity, oxidoreductase activity and antioxidant activity (Fig. 7b). After treatment with NaCl, a total of 1780 and 1752 genes were up- and down- regulated, respectively, in the MT plants relative to the WT (Additional file 1: Fig. S7b). The GO enrichment of the down-regulated genes in the MT plants indicated that they had decreased stress tolerance, which was consistent with the phenotype of MT under salt stress.

Using a 2-fold change in expression as the threshold, we analyzed the differentially expressed genes (DEGs) between the WT and MT. There were 1408 DEGs between the WT and MT lines under normal conditions, and 1150 DEGs between the lines under salt conditions (Fig. 7c). Among these genes, 162 were continuously up-regulated, and 142 were continuously down-regulated, in both conditions (Fig. 7c). We considered these genes to be regulated by OsNAC45. Heat maps show the genes that were differently expressed in the MT and WT lines after salt treatment, which may also be regulated by OsNAC45 (Additional file 1: Fig. S8a, b).

**Fig. 8:**
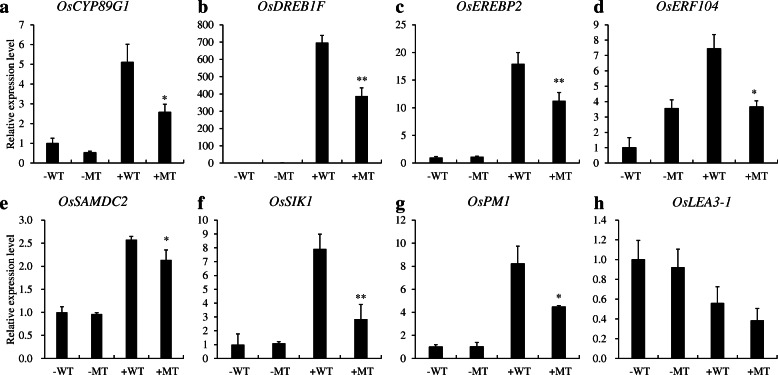
Relative expression levels of eight possible DEGs in MT (*osnac45*-knockout) and WT (Nipponbare) roots under normal conditions and high salinity. *OsCYP89G1* (**a**), *OsDREB1F* (**b**), *OsEREBP2* (**c**), *OsERF104* (**d**), *OsSAMDC2* (**e**), *OsSIK1* (**f**), *OsPM1* (**g**), *OsLEA3–1* (**h**). -WT and -MT indicate Nipponbare and *OsNAC45*-knockout lines under control conditions. +WT and + MT represent Nipponbare and *OsNAC45*-knockout lines treated with 100 mM NaCl for 24 h, respectively. Data are means ± SD of three biological replicates. *P < 0.05, **P < 0.01 by Student’s t test

To confirm these results, we performed qRT-PCR to check the expression profiles of some DEGs under both normal and salt conditions. As expected, we found that the changes in the expression levels of the DEGs under different conditions were not the same in the WT and MT plants. After salt treatment, the expression level of six genes was found to be lower in the MT lines than in WT plants including *OsCYP89G1* (Os07g0451300), *OsDREB1F* (Os01g0968800), *OsEREBP2* (Os01g0868000), *OsERF104* (Os08g0474000), *OsSAMDC2* (Os02g0611200), and *OsSIK1* (Os06g0130100) (Fig. 8a-f). Except for *OsERF104* and *OsCYP89G1*, other four genes have been reported to be involved in rice salt tolerance (Wang et al. 2008; Ouyang et al. 2010; Serra et al. 2013; Chen et al. 2014). These results indicate that *OsNAC45* probably affected these salt-tolerance related genes to regulate salt tolerance in rice.

Since previous studies hypothesized that *OsPM1* (Os05g0381400) and *OsLEA3–1* (Os05g0542500) may be regulated by OsNAC45 (Zheng et al. 2009), we performed qRT-PCR to check the expression profiles of these two genes. The results showed that salt treatment induced the expression of *OsPM1* by 8-fold in WT plants, but only by 4-fold in MT plants (Fig. 8g). However, there were no obvious differences in the expression profiles of *OsLEA3–1* in WT and MT plants under normal or salt conditions (Fig. 8h). These results suggest that *OsPM1,* but not *OsLEA3–1,* may be regulated by OsNAC45.

## Discussion

In this study, we found that ABA treatment decreased the expression of *OsNAC45*, but this effect was not dose-dependent (Fig. 1d). In the absence of ABA, the germination rates of the WT, *osnac45* and *OsNAC45*-overexpressed lines were similar, but the seeds of the overexpression lines germinated more slowly (Fig. 3b). A recent study demonstrated that overexpressing *OsNAC2* in transgenic rice enhances plant resistance to drought and salt stresses but causes a delay in seed germination (Jiang et al. 2019). This suggests that OsNAC45 and OsNAC2 use similar pathways in ABA signal transduction. After germinating, the shoots of the overexpression lines grew similarly to the WT plants (Fig. 4a, d). In the presence of ABA, the germination and growth of the *OsNAC45* overexpression seedlings were severely inhibited compared with the WT (Figs. 3a, c, d; 4b, c, e, f). On the contrary, the *osnac45* seedlings germinated more quickly and grew faster than the WT (Figs. 3a, c, d; 4b, c, e, f). These results demonstrate that OsNAC45 participates in ABA signaling pathways during the germination and early growth of rice.

Gene redundancy sometimes brings obstacles in biological studies: knock-outs or knock-downs of certain genes sometimes fail to provide informative phenotypes that may provide clue to the gene’s role. For example, mutant rice plants lacking *OsbZIP62* have no obvious mutant phenotypes in response to ABA treatment, whereas plants overexpressing this gene are hypersensitive to ABA (Yang et al. 2019). A previous study suggested that over-expressing *OsNAC45* enhances rice resistance to salt stress (Zheng et al. 2009). To further elucidate the role of OsNAC45 in rice, we used a CRISPR/Cas9 approach to generate *OsNAC45* knock-out lines. We found that the leaves of the *osnac45* lines were more withered and yellow compared with the WT after 75 mM or 100 mM NaCl (Fig. 5b, c). After 10 days of recovery, salt toxicity symptoms were still more noticeable in the *osnac45* lines (Fig. 5e, f). The differences in the root and shoot dry weights of WT and MT lines after the salt treatment and recovery period were consistent with their phenotypes observed (Additional file 1 Fig. S5). These results demonstrate that loss of *OsNAC45* makes rice more sensitive to salt stress, indicating that *OsNAC45* is required for salt tolerance in rice.

To find out how changing the expression of OsNAC45 affects the expression of its downstream genes, we performed an RNA-seq assay. The results showed that the expression levels of over 2000 genes were changed in MT lines under two different conditions (Fig. 7c). Under normal conditions, 2746 genes were down-regulated and gene ontology (GO) annotation analysis showed that these genes mainly belong to following categories: responses to oxidative stress, responses to stress, peroxidase activity, oxidoreductase activity and antioxidant activity (Fig. 7b). These results show that loss of *OsNAC45* down-regulates many stress-related genes, which may account for the increased sensitivity of the *osnac45* lines to salt stress. Among these down-regulated genes, we confirmed the expression levels of six of the down-regulated genes, *OsCYP89G1*, *OsDREB1F*, *OsEREBP2*, *OsERF104*, *OsSAMDC2*, and *OsSIK1*, by qRT-PCR. Previous reports found that *OsCYP89G1* and *OsERF104* belong to the ERF and CPY families, respectively, and may participate in stress responses, but their exact roles in response to salt stress remain unclear (Mishra et al. 2013; Wei and Chen 2018). *OsDREB1F* encoding a transcription factor was shown to enhance rice salt tolerance through activating the expression of some stress-related genes by specifically binding to DRE/CRT element (G/ACCGAC) (Wang et al. 2008). *OsEREBP2* was also a transcription factor and mediated rice salt tolerance through regulating a receptor-like kinase *OsRMC* which is a negative regulator of salt stress response (Serra et al. 2013). *OsSAMDC2* and *OsSIK1* encoded a S-adenosylmethionine decarboxylase gene and a stress-induced protein kinase gene, respectively. These two genes were proposed to be involved in rice salt tolerance through ROS scavenging pathway. Overexpression of *OsSIK1* could efficiently activate antioxidative system (Ouyang et al. 2010). Down-regulation of *OsSAMDC2* resulted in reduced many antioxidant enzyme activities (Chen et al. 2014). Therefore, more ROS accumulation in MT roots after salt treatment could be attributed to lower expression level of *OsSAMDC2* and *OsSIK1* compared with WT.

In a previous study, the expression levels of *OsPM1* (plasma membrane protein 1) and *OsLEA3–1* (late embryogenesis abundant) were found to be higher in transgenic rice plants overexpressing *OsNAC45* than in the WT (Zheng et al. 2009). However, when we measured the expression levels of these two genes in the MT and WT lines, our results indicated that *OsPM1*, but not *OsLEA3–1*, may be regulated by OsNAC45 (Fig. 8g, h). These findings suggest that *OsLEA3–1* expression may be controlled by some other genes that are up-regulated by OsNAC45, but not OsNAC45 itself.

Previous study has reported that over-expressing *OsNAC5* and *OsNAC6* in rice significantly improves plant salt stress resistance and that OsNAC5 and OsNAC6 regulate the expression of *OsLEA3–1* (Takasaki et al. 2010). This suggests that OsNAC45 is involved in different regulatory pathways to OsNAC5 or OsNAC6. *OsPM1* encodes a plasma membrane protein that mediates ABA influx through the plasma membrane (Yao et al. 2018). Additionally, OsNAC45 also regulates the expression of *OsDREB1F* (Fig. 8b), which is reported to be involved in ABA-dependent signal pathway (Wang et al. 2008). These may partially explain the phenotypes we observed in *OsNAC45* transgenic rice under ABA treatment.

## Conclusions

In conclusion, our results demonstrate that OsNAC45 plays important roles in ABA signal responses and is required for rice salt tolerance through regulating the expression of multiple stress-related genes*.*

## Methods

### Generation of Transgenic Plants

The CRISPR/Cas9 gene editing system was used according to the previously described protocol to generate transgenic *OsNAC45* knockout rice plants (Ma et al. 2015). To create a construct to overexpress *OsNAC45* in rice (named *OsNAC45*-OE), cDNA obtained from reverse transcription-PCR (RT-PCR) of Nipponbare total RNA was used as a template to obtain the coding region of *OsNAC45*. The full-length cDNA of *OsNAC45* was inserted into the pCAMBIA1300*-Ubi* vector carrying the maize *Ubiquitin* promoter and the terminator of the nopaline synthase gene. *OsNAC45*-OE and *OsNAC45*-knockout vectors were then introduced to *Agrobacterium tumefaciens* strain EHA101 and transformed into wild-type (WT) rice (*Oryza sativa* cv. Nipponbare). The primers used in this study were listed in Additional file 1 Table S1.

To detect the presence of the desired mutations in the rice plants, we performed DNA sequencing using *OsNAC45*-specific primers and the sequence chromatograms were aligned with the WT (*Oryza sativa* cv. Nipponbare) controls. Two homozygous mutants and two overexpressing *OsNAC45* lines were selected for further experiments.

### Plant Materials and Growth Conditions

The WT rice (*Oryza sativa* cv. Nipponbare) was used as a transformation recipient in this study. Rice seeds were soaked in water for 2 days and then the germinated seeds were put on a net floating on 0.5 mM CaCl_2_ solution in a greenhouse and used for various experiments.

For tissue specificity analysis, samples of root, stem, leaf, leaf sheath and spike were harvested from Nipponbare plants after the heading stage. For the salt treatment, 7-day-old seedlings of Nipponbare were exposed to different concentrations of NaCl (0, 50, 100, 150 and 200 mM) for 12 h or 100 mM NaCl for different times (3, 6, 12 and 24 h). For each condition, roots from 5 to 7 plants were collected at every timepoint for RNA extraction. For the ABA treatment, 7-day-old Nipponbare seedlings were exposed to different concentrations of ABA (0, 6, 12, 30, 50 and 100 μM) for 6 h. For each condition, roots of 5–7 plants were sampled at every timepoint for RNA extraction.

### RNA Isolation and Gene Expression Analysis

Rice RNA was extracted with the Trizol reagent kit (Life technologies, USA) according to the manufacturer’s instructions. First-strand cDNA was synthesized using a Hiscript II Q RT SuperMix Kit (Vazyme) with 1 μg total RNA. Quantitative reverse transcription-PCR (qRT-PCR) was performed with ChanQTM SYBR Color qPCR Master Mix (Vazyme) on a StepOnePlus Real-Time PCR System (Analytik Jena AG). *Histone H3* was used as an internal standard. The relative expression levels of the genes were calculated by the 2^-ΔΔCT^ method.

### Transactivation Activity Assay

The full-length coding region of *OsNAC45* was amplified by PCR and fused with the GAL4 DNA-binding domain in a pGBKT7 vector. AH109 yeast cells transformed with GBK-OsNAC45 or a control construct were spotted on SD/Trp- and SD/Trp−/His−/Ade- medium and incubated at 30 °C.

### Subcellular Localization of OsNAC45

The subcellular localization of OsNAC45 was investigated in rice protoplasts by fusing OsNAC45 to green fluorescent protein (GFP). Full-length cDNA of *OsNAC45* was cloned into the pYL322-*GFP* vector after the *GFP* coding region to make the *GFP*-*OsNAC45* vector.

A nucleus marker (*GFP-OsGhd7*) plus *GFP*-*OsNAC45* or the control pYL322-*GFP* vector were transformed into rice protoplasts with PEG as previously described (Chen et al. 2011). The protoplasts were placed under a confocal laser scanning microscope to gather images of the GFP fluorescence (TCS SP8; Leica).

### Cellular Localization of OsNAC45

The promoter (2157 bp) of *OsNAC45* was amplified by PCR from Nipponbare genomic DNA, and the coding region of *OsNAC45* without the stop codon was amplified by PCR from cDNA extracted from Nipponbare roots. To make a construct encoding the OsNAC45 protein fused to GFP at its N-terminal end, the amplified fragment was cloned into the pCAMBIA1300-*GFP* vector. The resulting construct (named *ProOsNAC45*-*OsNAC45*-*GFP*) was transformed into Nipponbare, producing transgenic lines carrying *ProOsNAC45-OsNAC45-GFP*.

To investigate the cellular localization of OsNAC45, immunostaining was performed using an antibody against GFP as previously described (Yamaji and Ma 2007). Briefly, the roots of WT and transgenic lines carrying *ProOsNAC45-OsNAC45-GFP* were embedded in 5% agar and sectioned 100-μm thick with a microslicer (VT1000 S, Leica). Sections were placed on microscope slides and incubated with the rabbit anti-GFP polyclonal antibodies and secondary antibodies (Alexa Fluor 555 goat anti-rabbit IgG; Molecular Probes) at room temperature, respectively. Then sections were stained with DAPI. A confocal laser scanning microscope was used to collect fluorescence images (TCS SP8; Leica, wetzlar, Germany).

### Germination Assay and Young Seedling Growth Assay

For the germination assays, rice seeds (30 seeds for each line) were placed on 1/2 MS medium contain 0, 1, and 2 μM ABA (purity> 99%, Biotopped) at 28 °C, and the number of germinated seeds were counted every 12 h for 5 days.

For the young seedling growth assay, rice seeds (30 seeds for each line) were placed on 1/2 MS medium at 28 °C for 2 days, and plants of a similar size were transferred to a plastic box (15 × 11 × 6 cm) containing 1/2 MS medium containing 0, 1 or 2 μM ABA. Shoot length was measured 5 days after the transfer.

### Determination of Salt Tolerance and Element Concentration

We exposed 30-day-old seedlings of WT (Nipponbare) and *OsNAC45*-knockout lines (*osnac45–14*, *osnac45–15*) to 0, 75 or 100 mM NaCl for 10 days and then returned the plants to their normal growth conditions for 10 days. After the treatment and recovery time, roots and shoots of the WT and *osnac45* were sampled and dehydrated at 70 °C for 3 days and then the dry weights of the samples were measured. The dried samples were digested in boiling tubes with 65% HNO_3_. The Na^+^ and K^+^ concentrations in the digested solution were determined by ICP-MS (Plasma Quant MS; Analytik Jena AG).

### NBT Staining

We exposed 5-day-old seedlings of WT (Nipponbare) and *OsNAC45*-knockout lines (*osnac45–14*, *osnac45–15*) to 0 or 100 mM NaCl for 24 h. The roots of all lines were incubated with 6 mM NBT for 10 min. Then roots were embedded in low melting agarose and sectioned at 5 mm from root tip (100-μm thickness) using a slicer (VT1000 S, Leica). Sections were photographed by a Nikon E100 microscope.

### RNA-seq Experiments

Two-week-old seedlings of Nipponbare and the *OsNAC45* mutant lines (MT) were exposed to 100 mM NaCl for 24 h and then the roots of seedlings were sampled for further experiments. Total RNA was extracted and purified to obtain the total mRNA. Then, we synthesized the first strand cDNA and second strand cDNA using the reverse transcriptase and DNA polymerase enzymes, respectively. After constructing the cDNA ibrary, RNA-sequencing was carried out on an Illumina NovaSeq platform. Deseq2 was used to analyze the differences in gene expression between the WT and MT lines. The DEGs (differentially expressed genes) were selected by the standard of |log2_ratio| > 2. GO (Gene Ontology; http://geneontology.org/) analysis of DEGs was carried out by hypergeometric tests, and each *p*-value indicates the enrichment of the corresponding category.

## Supplementary Information

**Additional file 1: Figure S1.** Transactivation assay of OsNAC45. The full-length CDS of *OsNAC45* was fused to GAL4 binding domain (BD) and transformed into yeast strain AH109, vector pGBKT7 was used as control. The transformants were placed on media containing SD/Trp- (**a**) and SD/Trp−/His−/Ade- (**b**). **Figure S2.** Subcellular localization of OsNAC45 in rice protoplasts. The upper panels show the cells co-expressing GFP and a nuclear marker OsGhd7 under the control of the CaMV35S promoter. The lower panels show the cells co-expressing GFP-OsNAC45 and a nuclear marker Ghd7 under the control of the CaMV35S promoter. Scale bar = 10 μm. **Figure S3.** OsNAC45 sequence of two independent mutants generated by CRISPR/Cas9 mutagenesis. Black box indicates the exon. **Figure S4.** Relative expression levels of *OsNAC45* in the *OsNAC45*-overexpression lines (OE-10, OE-13). Data are means ± SD of three biological replicates. **Figure S5** *OsNAC45* knock-out mutants are more sensitive to salt stress. Dry weights of shoots and roost in WT and *osnac45* lines treated with 0 mM (**a**), 75 mM (**b**), 100 mM (**c**) NaCl for 10 days. Dry weights of shoots and roots in WT and *osnac45* lines that were allowed to recover for 10 days after the 10-day treatment with 0 mM (**d**), 75 mM (**e**), 100 mM (**f**) NaCl. The data represent the means ± SD (*n* = 4 each), **P < 0.01, *P < 0.05 according to the Student’s t test. **Figure S6.** Tissue Na^+^ and K^+^ concentration in WT and *OsNAC45* knockout lines under normal condition or 100 mM NaCl treatment for 10 days. a: Shoot Na^+^ concentration. b: Shoot K^+^ concentration. c: Root Na^+^ concentration. d: Root K^+^ concentration. **Figure S7.** Differently expressed genes in the roots of MT plants. (**a**) Up- and down- regulated genes in the roots of MT compared with WT under normal conditions. (**b**) Up- and down- regulated genes in the roots of MT compared with WT in high salinity. Green dots indicate down-regulated genes, red dots indicate up-regulated genes, blue dots indicate other genes whose expression did not change. Three biological replicates (*n* = 3) were performed in each treatment. **Figure S8.** Cluster analysis of the OsNAC45-regulated genes after salt treatment. Genes downregulated (**a**) or upregulated (**b**) after salt treatment. A1–3 represents three biological replicates of WT, F1–3 represent three biological replicates of MT. **Table S1**. Primers used in this study.

**Additional file 2:**

## Data Availability

All data supporting the conclusions of this article are provided within the article (and its additional files).

## Abbreviations

ABA: Abscisic acid
NAC: NAM, AFAT, and CUC transcription factor
qRT-PCR: Reverse transcription quantitative PCR
WT: Wild-type
MT: Mutant
OE: Overexpression
GFP: Green Fluorescent Protein
ROS: Reactive oxygen species
NBT: Nitroblue tetrazolium

## References

[CR1] Aida M, Ishida T, Fukaki H, Fujisawa H, Tasaka M (1997). Genes involved in organ separation in Arabidopsis: an analysis of the cup-shaped cotyledon mutant. Plant Cell.

[CR2] Chen J, Liu Y, Ni J, Wang Y, Bai Y, Shi J, Gan J, Wu Z, Wu P (2011). OsPHF1 regulates the plasma membrane localization of low- and high-affinity inorganic phosphate transporters and determines inorganic phosphate uptake and translocation in rice. Plant Physiol.

[CR3] Chen M, Chen J, Fang J, Guo Z, Lu SJPCT, Culture O (2014). Down-regulation of S-adenosylmethionine decarboxylase genes results in reduced plant length, pollen viability, and abiotic stress tolerance. Plant Cell Tissue Organ Cult.

[CR4] Dai X, Xu Y, Ma Q, Xu W, Wang T, Xue Y, Chong K (2007). Overexpression of an R1R2R3 MYB gene, OsMYB3R-2, increases tolerance to freezing, drought, and salt stress in transgenic Arabidopsis. Plant Physiol.

[CR5] Fujita M, Fujita Y, Maruyama K, Seki M, Hiratsu K, Ohme-Takagi M, Tran LS, Yamaguchi-Shinozaki K, Shinozaki K (2004). A dehydration-induced NAC protein, RD26, is involved in a novel ABA-dependent stress-signaling pathway. Plant J.

[CR6] Gao F, Xiong AS, Peng RH, Jin XF, Xu J, Zhu B, Chen JM, Yao QH (2010). OsNAC52, a rice NAC transcription factor, potentially responds to ABA and confers drought tolerance in transgenic plants. Plant Cell Tissue Org Cult.

[CR7] Hong Y, Zhang H, Huang L, Li D, Song F (2016). Overexpression of a stress-responsive NAC transcription factor gene ONAC022 improves drought and salt tolerance in rice. Front Plant Sci.

[CR8] Hu H, Dai M, Yao J, Xiao B, Li X, Zhang Q, Xiong L (2006). Overexpressing a NAM, ATAF, and CUC (NAC) transcription factor enhances drought resistance and salt tolerance in rice. Proc Natl Acad Sci U S A.

[CR9] Hu H, You J, Fang Y, Zhu X, Qi Z, Xiong L (2008). Characterization of transcription factor gene SNAC2 conferring cold and salt tolerance in rice. Plant Mol Biol.

[CR10] Huh SU, Lee SB, Kim HH, Paek KH (2012). ATAF2, a NAC transcription factor, binds to the promoter and regulates NIT2 gene expression involved in auxin biosynthesis. Mol Cells.

[CR11] Ismail AM, Horie T (2017). Genomics, physiology, and molecular breeding approaches for improving salt tolerance. Annu Rev Plant Biol.

[CR12] Jeong JS, Kim YS, Baek KH, Jung H, Ha SH, Do Choi Y, Kim M, Reuzeau C, Kim JK (2010). Root-specific expression of OsNAC10 improves drought tolerance and grain yield in rice under field drought conditions. Plant Physiol.

[CR13] Jeong JS, Kim YS, Redillas MC, Jang G, Jung H, Bang SW, Choi YD, Ha SH, Reuzeau C, Kim JK (2013). OsNAC5 overexpression enlarges root diameter in rice plants leading to enhanced drought tolerance and increased grain yield in the field. Plant Biotechnol J.

[CR14] Jiang D, Zhou L, Chen W, Ye N, Xia J, Zhuang C (2019). Overexpression of a microRNA-targeted NAC transcription factor improves drought and salt tolerance in Rice via ABA-mediated pathways. Rice (N Y).

[CR15] Kim S, Kang JY, Cho DI, Park JH, Kim SY (2004). ABF2, an ABRE-binding bZIP factor, is an essential component of glucose signaling and its overexpression affects multiple stress tolerance. Plant J.

[CR16] Liu Q, Kasuga M, Sakuma Y, Abe H, Miura S, Yamaguchi-Shinozaki K, Shinozaki K (1998). Two transcription factors, DREB1 and DREB2, with an EREBP/AP2 DNA binding domain separate two cellular signal transduction pathways in drought- and low-temperature-responsive gene expression, respectively, in Arabidopsis. Plant Cell.

[CR17] Ma X, Zhang Q, Zhu Q, Liu W, Chen Y, Qiu R, Wang B, Yang Z, Li H, Lin Y, Xie Y, Shen R, Chen S, Wang Z, Chen Y, Guo J, Chen L, Zhao X, Dong Z, Liu YG (2015). A robust CRISPR/Cas9 system for convenient, high-efficiency multiplex genome editing in monocot and dicot plants. Mol Plant.

[CR18] Mao C, Lu S, Lv B, Zhang B, Shen J, He J, Luo L, Xi D, Chen X, Ming F (2017). A rice NAC transcription factor promotes leaf senescence via ABA biosynthesis. Plant Physiol.

[CR19] Mishra M, Kanwar P, Singh A, Pandey A, Kapoor S, Pandey GK (2013). Plant omics: genome-wide analysis of ABA repressor1 (ABR1) related genes in rice during abiotic stress and development. OMICS.

[CR20] Nakashima K, Tran LS, Van Nguyen D, Fujita M, Maruyama K, Todaka D, Ito Y, Hayashi N, Shinozaki K, Yamaguchi-Shinozaki K (2007). Functional analysis of a NAC-type transcription factor OsNAC6 involved in abiotic and biotic stress-responsive gene expression in rice. Plant J.

[CR21] Nambara E, Marion-Poll A (2005). Abscisic acid biosynthesis and catabolism. Annu Rev Plant Biol.

[CR22] Nuruzzaman M, Manimekalai R, Sharoni AM, Satoh K, Kondoh H, Ooka H, Kikuchi S (2010). Genome-wide analysis of NAC transcription factor family in rice. Gene.

[CR23] Ouyang SQ, Liu YF, Liu P, Lei G, He SJ, Ma B, Zhang WK, Zhang JS, Chen SY (2010). Receptor-like kinase OsSIK1 improves drought and salt stress tolerance in rice (Oryza sativa) plants. Plant J.

[CR24] Puranik S, Sahu PP, Srivastava PS, Prasad M (2012). NAC proteins: regulation and role in stress tolerance. Trends Plant Sci.

[CR25] Riechmann JL, Meyerowitz EM (1998). The AP2/EREBP family of plant transcription factors. Biol Chem.

[CR26] Sachs MM, Ho THD (1986). Alteration of gene expression during environmental stress in plants. Annu Rev Plant Physiol.

[CR27] Sakuraba Y, Piao W, Lim JH, Han SH, Kim YS, An G, Paek NC (2015). Rice ONAC106 inhibits leaf senescence and increases salt tolerance and tiller angle. Plant Cell Physiol.

[CR28] Serra TS, Figueiredo DD, Cordeiro AM, Almeida DM, Lourenço T, Abreu IA, Sebastián A, Fernandes L, Contreras-Moreira B, Oliveira MM, Saibo NJ (2013). OsRMC, a negative regulator of salt stress response in rice, is regulated by two AP2/ERF transcription factors. Plant Mol Biol.

[CR29] Shim JS, Oh N, Chung PJ, Kim YS, Choi YD, Kim JK (2018). Overexpression of OsNAC14 improves drought tolerance in rice. Front Plant Sci.

[CR30] Souer E, van Houwelingen A, Kloos D, Mol J, Koes R (1996). The no apical meristem gene of Petunia is required for pattern formation in embryos and flowers and is expressed at meristem and primordia boundaries. Cell.

[CR31] Sperotto RA, Ricachenevsky FK, Duarte GL, Boff T, Lopes KL, Sperb ER, Grusak MA, Fett JP (2009). Identification of up-regulated genes in flag leaves during rice grain filling and characterization of OsNAC5, a new ABA-dependent transcription factor. Planta.

[CR32] Takasaki H, Maruyama K, Kidokoro S, Ito Y, Fujita Y, Shinozaki K, Yamaguchi-Shinozaki K, Nakashima K (2010). The abiotic stress-responsive NAC-type transcription factor OsNAC5 regulates stress-inducible genes and stress tolerance in rice. Mol Gen Genomics.

[CR33] Tang N, Zhang H, Li X, Xiao J, Xiong L (2012). Constitutive activation of transcription factor OsbZIP46 improves drought tolerance in rice. Plant Physiol.

[CR34] VanWallendael A, Soltani A, Emery NC, Peixoto MM, Olsen J, Lowry DB (2019). A molecular view of plant local adaptation: incorporating stress-response networks. Annu Rev Plant Biol.

[CR35] Wang Q, Guan Y, Wu Y, Chen H, Chen F, Chu C (2008). Overexpression of a rice OsDREB1F gene increases salt, drought, and low temperature tolerance in both Arabidopsis and rice. Plant Mol Biol.

[CR36] Wei K, Chen H (2018). Global identification, structural analysis and expression characterization of cytochrome P450 monooxygenase superfamily in rice. BMC Genomics.

[CR37] Xie Q, Frugis G, Colgan D, Chua NH (2000). Arabidopsis NAC1 transduces auxin signal downstream of TIR1 to promote lateral root development. Genes Dev.

[CR38] Yamaji N, Ma JF (2007). Spatial distribution and temporal variation of the rice silicon transporter Lsi1. Plant Physiol.

[CR39] Yang S, Xu K, Chen S, Li T, Xia H, Chen L, Liu H, Luo L (2019). A stress-responsive bZIP transcription factor OsbZIP62 improves drought and oxidative tolerance in rice. BMC Plant Biol.

[CR40] Yao L, Cheng X, Gu Z, Huang W, Li S, Wang L, Wang Y-F, Xu P, Ma H, Ge X (2018). The AWPM-19 family protein OsPM1 mediates abscisic acid influx and drought response in rice. Plant Cell.

[CR41] Yu SW, Huang AN, Li J, Gao L, Feng YN, Pemberton E, Chen CL (2018). OsNAC45 plays complex roles by mediating POD activity and the expression of development-related genes under various abiotic stresses in rice root. Plant Growth Regul.

[CR42] Yuan X, Wang H, Cai J, Bi Y, Li D, Song F (2019). Rice NAC transcription factor ONAC066 functions as a positive regulator of drought and oxidative stress response. BMC Plant Biol.

[CR43] Zheng X, Chen B, Lu G, Han B (2009). Overexpression of a NAC transcription factor enhances rice drought and salt tolerance. Biochem Biophys Res Commun.

[CR44] Zhu JK (2002). Salt and drought stress signal transduction in plants. Annu Rev Plant Biol.

