# Supplementary material for: OsNAC45 is Involved in ABA Response and Salt Tolerance in Rice
Source: Rice (N Y). 2020 Dec 7;13:79. doi: 10.1186/s12284-020-00440-1 (PMC7721851; doi:10.1186/s12284-020-00440-1)
Supplement: Supplementary file 1 — Additional file 1: Figure S1. Transactivation assay of OsNAC45. The full-length CDS of OsNAC45 was fused to GAL4 binding domain (BD) and transformed into yeast strain AH109, vector pGBKT7 was used as control. The transformants were placed on media containing SD/Trp- (a) and SD/Trp−/His−/Ade- (b). Figure S2. Subcellular localization of OsNAC45 in rice protoplasts. The upper panels show the cells co-expressing GFP and a nuclear marker OsGhd7 under the control of the CaMV35S promoter. The lower panels show the cells co-expressing GFP-OsNAC45 and a nuclear marker Ghd7 under the control of the CaMV35S promoter. Scale bar = 10 μm. Figure S3. OsNAC45 sequence of two independent mutants generated by CRISPR/Cas9 mutagenesis. Black box indicates the exon. Figure S4. Relative expression levels of OsNAC45 in the OsNAC45-overexpression lines (OE-10, OE-13). Data are means ± SD of three biological replicates. Figure S5 OsNAC45 knock-out mutants are more sensitive to salt stress. Dry weights of shoots and roost in WT and osnac45 lines treated with 0 mM (a), 75 mM (b), 100 mM (c) NaCl for 10 days. Dry weights of shoots and roots in WT and osnac45 lines that were allowed to recover for 10 days after the 10-day treatment with 0 mM (d), 75 mM (e), 100 mM (f) NaCl. The data represent the means ± SD (n = 4 each), **P < 0.01, *P < 0.05 according to the Student’s t test. Figure S6. Tissue Na+ and K+ concentration in WT and OsNAC45 knockout lines under normal condition or 100 mM NaCl treatment for 10 days. a: Shoot Na+ concentration. b: Shoot K+ concentration. c: Root Na+ concentration. d: Root K+ concentration. Figure S7. Differently expressed genes in the roots of MT plants. (a) Up- and down- regulated genes in the roots of MT compared with WT under normal conditions. (b) Up- and down- regulated genes in the roots of MT compared with WT in high salinity. Green dots indicate down-regulated genes, red dots indicate up-regulated genes, blue dots indicate other genes whose expression did not [file 12284_2020_440_MOESM1_ESM.docx]

**Additional file 1:**


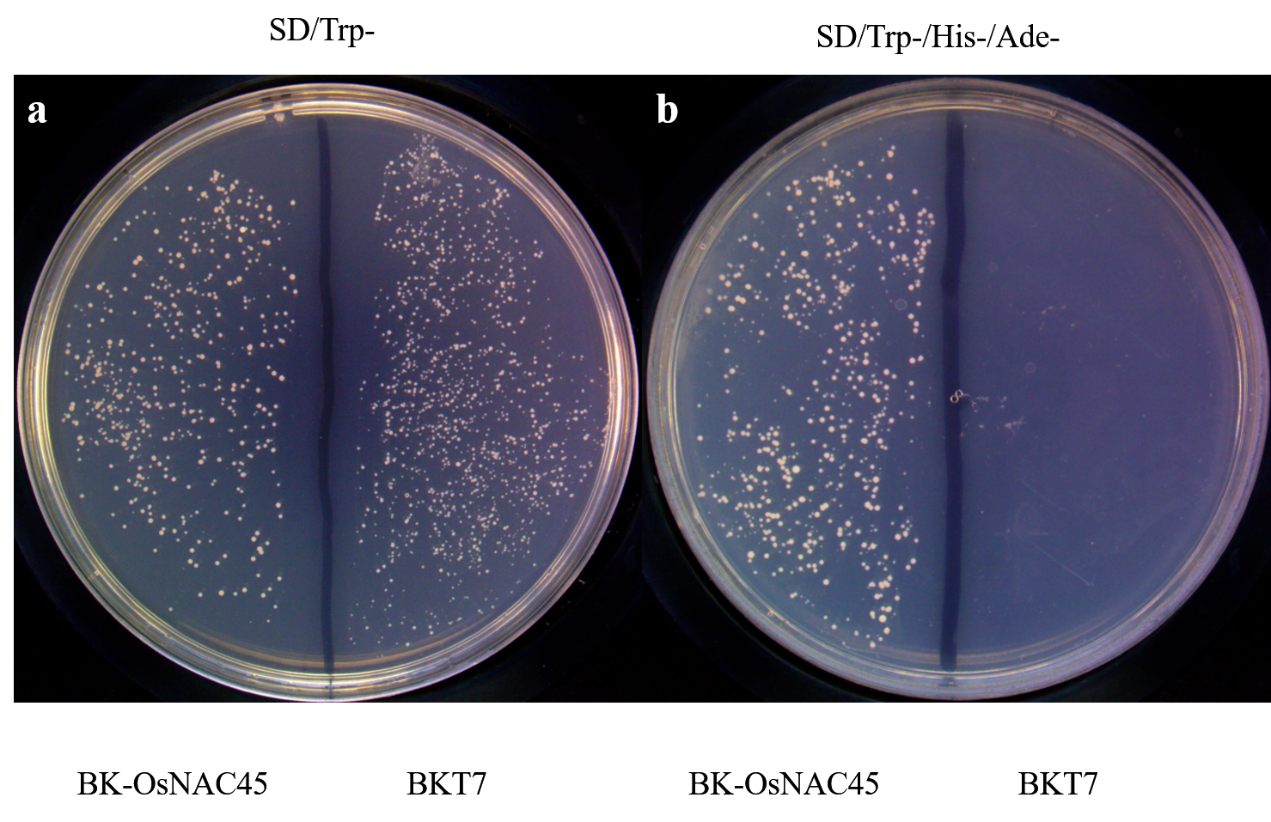


**Figure S1.** Transactivation assay of OsNAC45. The full-length CDS of *OsNAC45* was fused to GAL4 binding domain (BD) and transformed into yeast strain AH109, vector pGBKT7 was used as control. The transformants were placed on media containing SD/Trp- (**a**) and SD/Trp-/His-/Ade- (**b**).


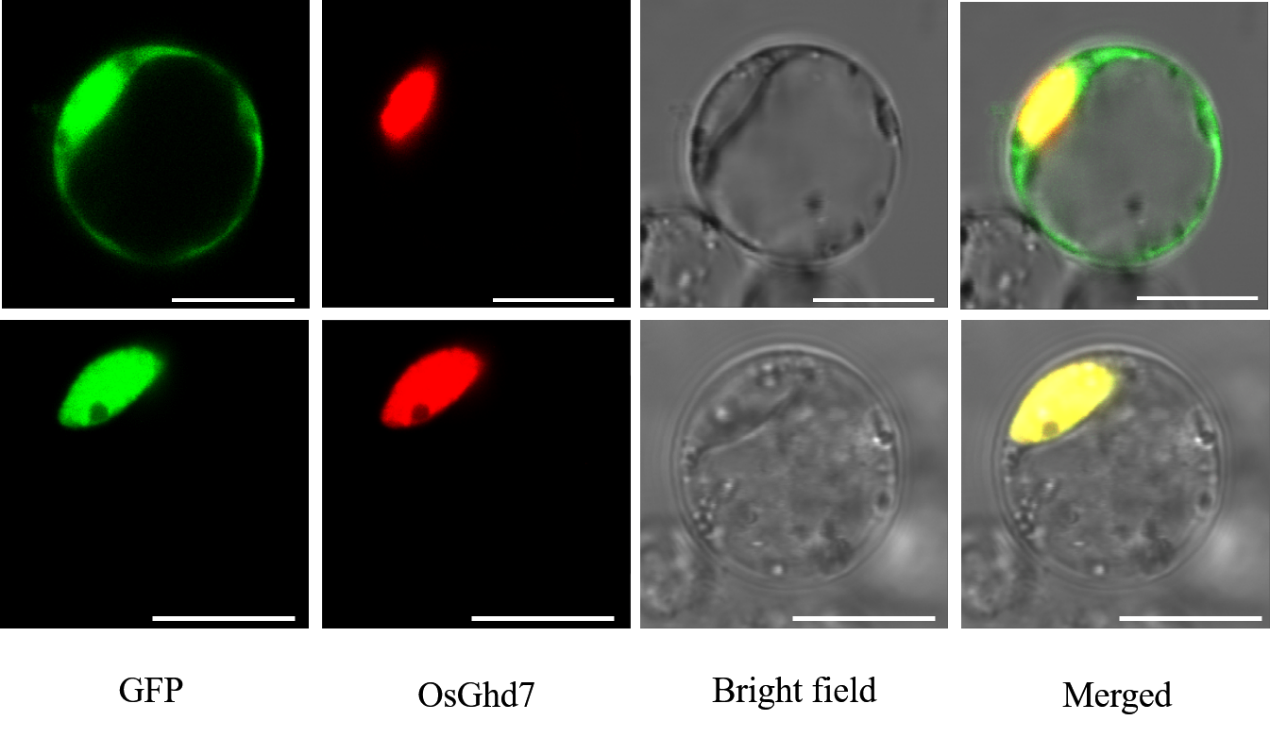


**Figure S2.** Subcellular localization of OsNAC45 in rice protoplasts. The upper panels show the cells co-expressing GFP and a nuclear marker OsGhd7 under the control of the CaMV35S promoter. The lower panels show the cells co-expressing GFP-OsNAC45 and a nuclear marker Ghd7 under the control of the CaMV35S promoter. Scale bar = 10 μm.


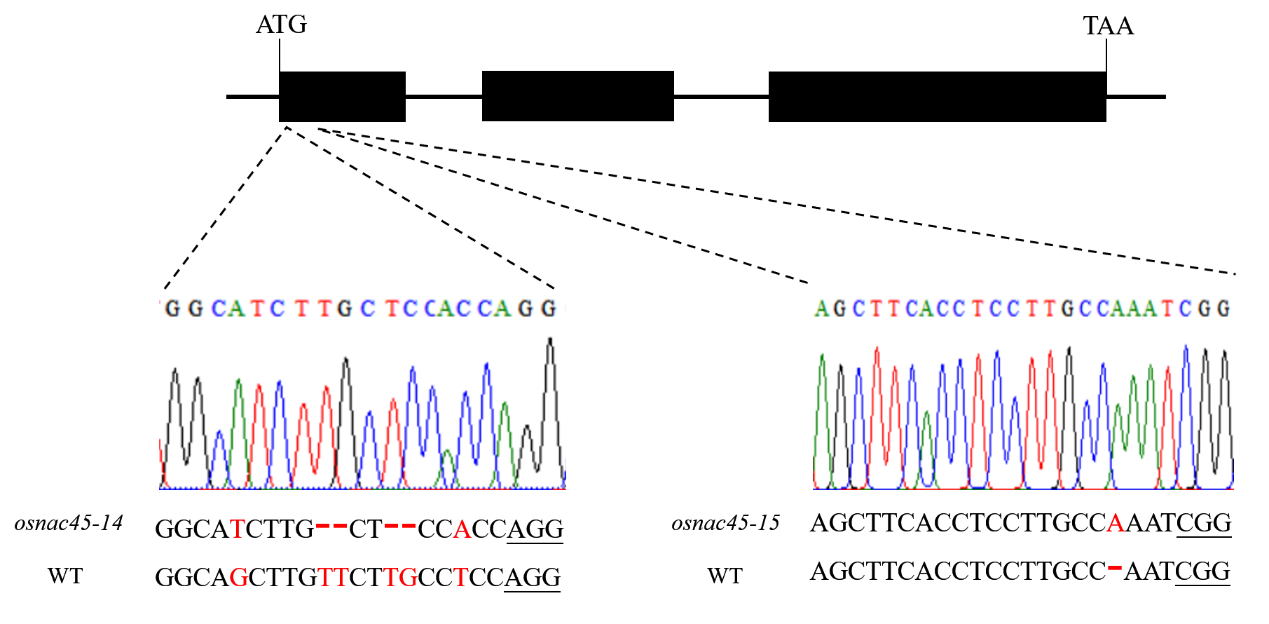


**Figure S3.** OsNAC45 sequence of two independent mutants generated by CRISPR/Cas9 mutagenesis. Black box indicates the exon.


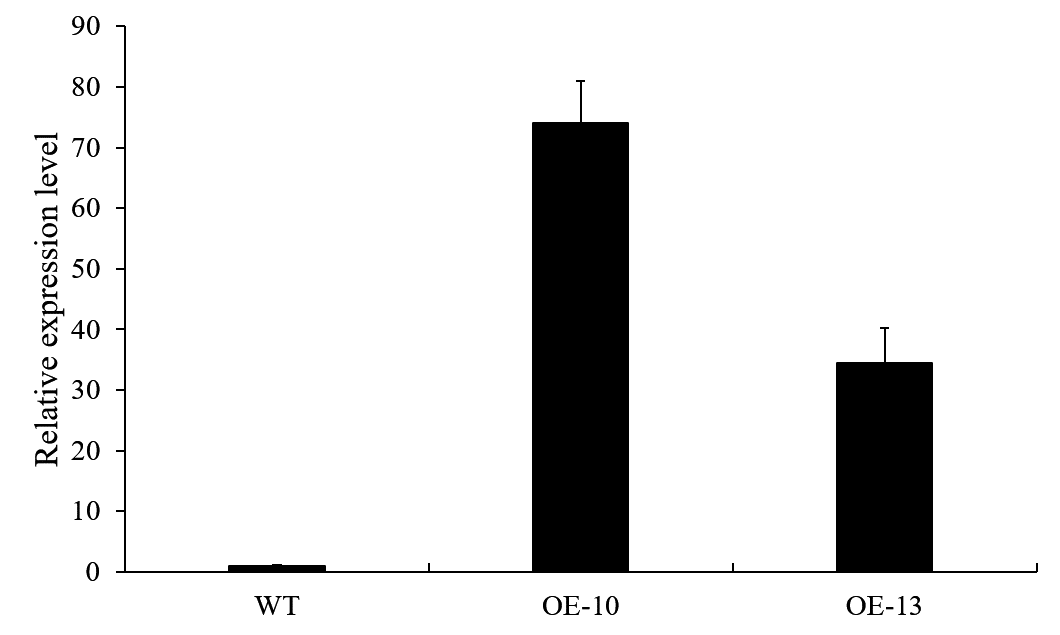


**Figure S4.** Relative expression levels of *OsNAC45* in the *OsNAC45*-overexpression lines (OE-10, OE-13). Data are means ± SD of three biological replicates.


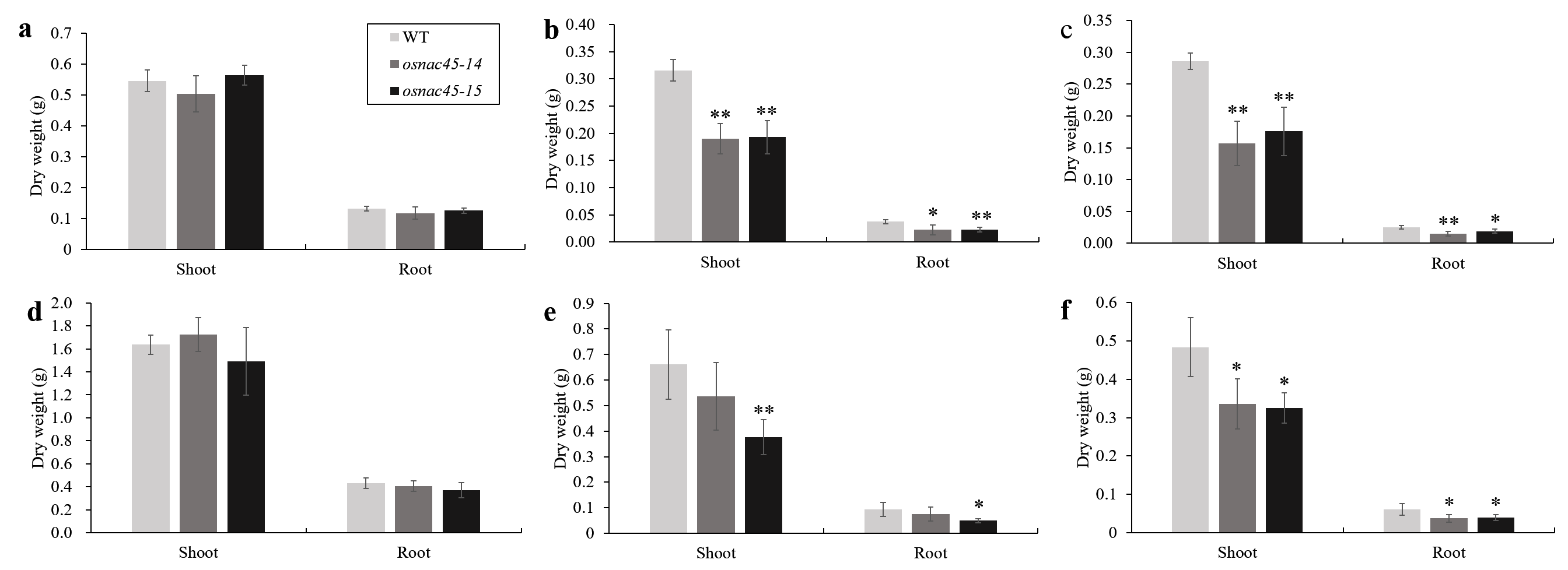


**Figure S5** *OsNAC45* knock-out mutants are more sensitive to salt stress. Dry weights of shoots and roost in WT and *osnac45* lines treated with 0 mM (**a**), 75 mM (**b**), 100 mM (**c**) NaCl for 10 days. Dry weights of shoots and roots in WT and *osnac45* lines that were allowed to recover for 10 days after the 10-day treatment with 0 mM (**d**), 75 mM (**e**), 100 mM (**f**) NaCl. The data represent the means ± SD (n = 4 each), **P < 0.01, *P < 0.05 according to the Student’s t test.


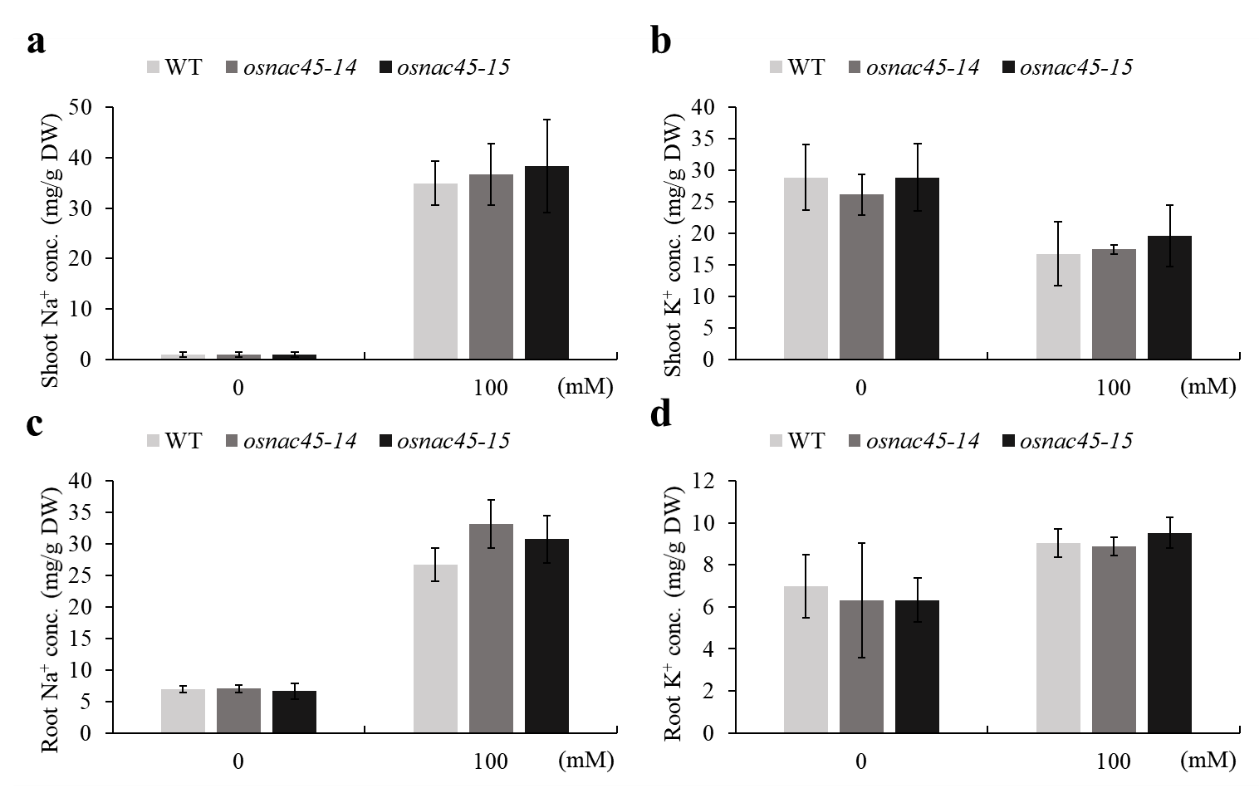


**Figure S6.** Tissue Na^+^ and K^+^ concentration in WT and *OsNAC45* knockout lines under normal condition or 100 mM NaCl treatment for 10 days. a: Shoot Na^+^ concentration. b: Shoot K^+^ concentration. c: Root Na^+^ concentration. d: Root K^+^ concentration.


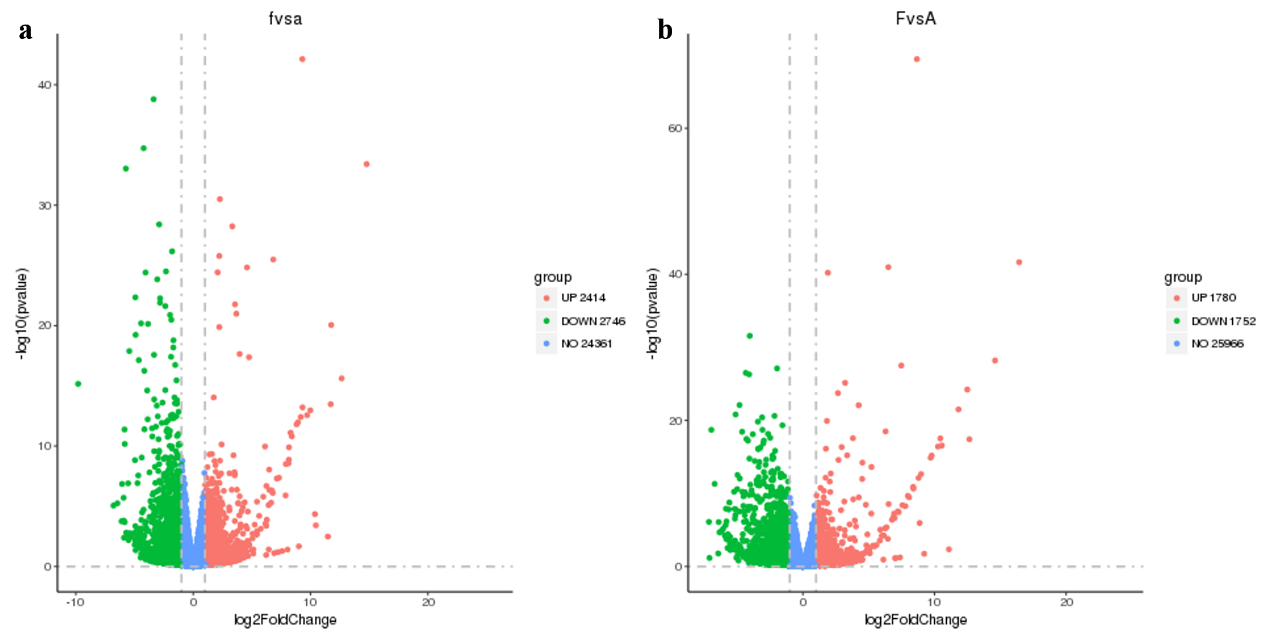


**Figure S7.** Differently expressed genes in the roots of MT plants. (**a**) Up- and down- regulated genes in the roots of MT compared with WT under normal conditions. (**b**) Up- and down- regulated genes in the roots of MT compared with WT in high salinity. Green dots indicate down-regulated genes, red dots indicate up-regulated genes, blue dots indicate other genes whose expression did not change. Three biological replicates (n = 3) were performed in each treatment.


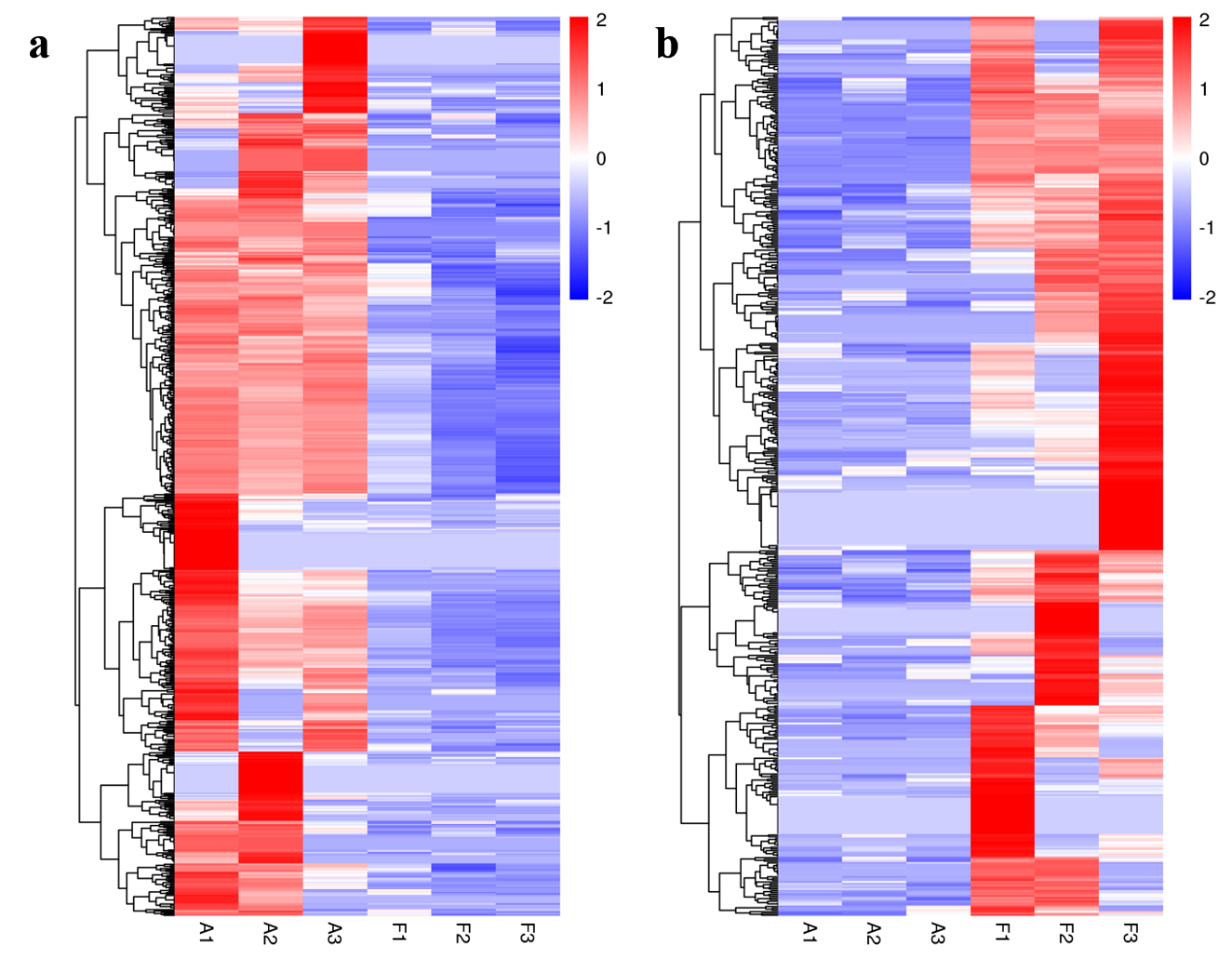


**Figure S8.** Cluster analysis of the OsNAC45-regulated genes after salt treatment. Genes downregulated (**a**) or upregulated (**b**) after salt treatment. A1–3 represents three biological replicates of WT, F1–3 represent three biological replicates of MT.


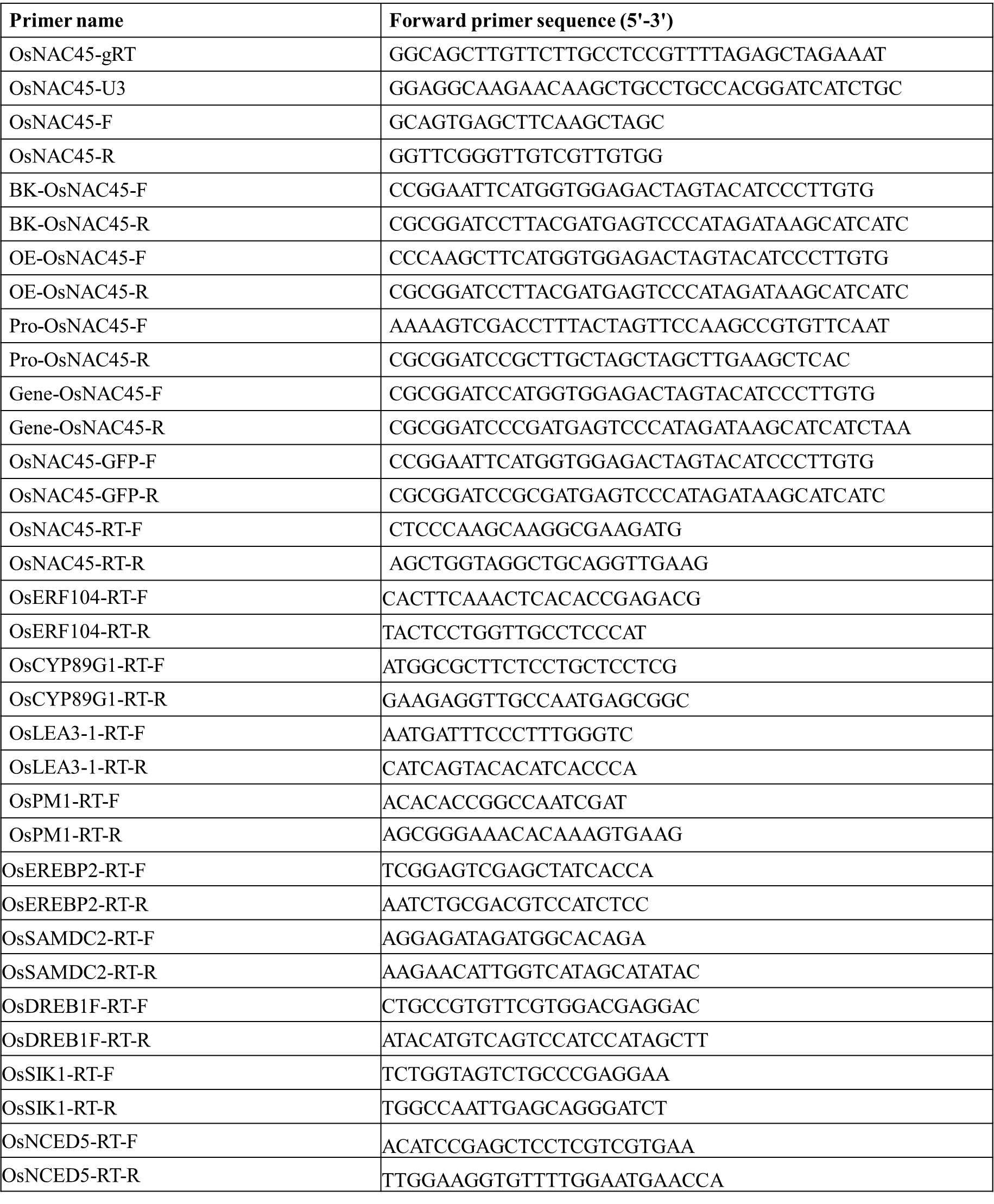


Table S1: Primers used in this study
